# 460. Disproportionalities in COVID-19 Clinical Drug Trials in the United States: A Systematic Literature Review

**DOI:** 10.1093/ofid/ofab466.659

**Published:** 2021-12-04

**Authors:** Daniel B Chastain, Vishwa S Patel, Alexandria M Jefferson, Sharmon P Osae, Joeanna S Chastain, Andrés F Henao Martínez, Carlos Franco-Paredes, Henry N Young

**Affiliations:** 1 University of Georgia College of Pharmacy, Albany, GA; 2 University of Georgia, Athens, Georgia; 3 Phoebe Putney Memorial Hospital, Albany, Georgia; 4 University of Colorado Anschutz Medical Campus, Aurora, Colorado; 5 University of Colorado Denver, School of Medicine, Aurora, CO

## Abstract

**Background:**

To combat higher rates of COVID-19 infection, hospitalization, and death among minorities, it is crucial to identify safe, efficacious, and generalizable treatments. Therefore, the purpose of this systematic literature review was to assess the demographic characteristics of COVID-19 clinical trial participants.

**Methods:**

A literature search was performed according to the PRISMA checklist using PubMed from December 1, 2019 to November 24, 2020 with the following search terms: 2019-nCoV, COVID-19, SARS-CoV-2, clinical trial, randomized controlled trial, observational study, and veterinary. To capture additional results, keyword searches were performed using various versions and plural endings with the title/abstract field tag. Randomized controlled trials evaluating a pharmacologic treatment for COVID-19 patients from one or more U.S site written in the English language were eligible for inclusion. Descriptive statistics were calculated to characterize age, gender, race, and ethnicity of patients enrolled in the included COVID-19 clinical trials, as well as for comparison with national COVID-19 data.

**Results:**

A total of 4472 records were identified, of which 16 were included. Most were placebo-controlled (69%) and included hospitalized patients with COVID-19 (69%). Demographic data were reported for each study arm in 81% of studies. Median number of participants was higher in studies of nonhospitalized patients (n=452 [range 20-1062] vs n=243 [range 152-2795]). Nine (56%) studies reported mean or median ages of 50 years or older amongst all study arms. Males comprised more than half of the study cohort in 50% of studies. Race and ethnicity were reported separately in five (31%) studies, reported in combination in four (25%), while six (38%) reported only race or ethnicity. White or Caucasian patients made up most participants across all arms in 75% of studies. Based on national COVID-19 data, hospitalizations were similar between White persons and African American persons, but higher than other race or ethnic groups, and evenly distributed among males and females.

**Conclusion:**

Lack of heterogeneously reporting demographic characteristics of COVID-19 clinical trial participants limits the ability to assess the generalizability of pharmacologic treatments for COVID-19.

**Disclosures:**

**All Authors**: No reported disclosures

